# Monophosphoryl Lipid A and Poly I:C Combination Adjuvant Promoted Ovalbumin-Specific Cell Mediated Immunity in Mice Model

**DOI:** 10.3390/biology10090908

**Published:** 2021-09-13

**Authors:** So Yeon Ahn, Chau Thuy Tien Le, Eun-Ju Ko

**Affiliations:** 1 Department of Veterinary Medicine, College of Veterinary Medicine, Jeju National University, Jeju 63243, Korea; asy0078@gmail.com; 2 Interdisciplinary Graduate Program in Advanced Convergence Technology & Science, Jeju National University, Jeju 63243, Korea; thuytienle102@gmail.com

**Keywords:** cell-mediated immunity, memory T cell, dendritic cell, monophosphoryl lipid A, Poly I:C

## Abstract

**Simple Summary:**

Many research groups have investigated and developed new adjuvant candidates to promote vaccine efficacy, but only few of them were licensed. A combination of toll-like receptor (TLR) agonists can be a promising vaccine adjuvant candidate by stimulating innate immune cells and inducing antigen-specific cell-mediated immunity (CMI). In this study, a monophosphoryl lipid A (MPL) and Poly I:C combination, in low doses with ovalbumin (OVA) protein, elicited strong OVA-specific antibody production and more effective memory T cell responses compared with OVA only, OVA+MPL, OVA+Poly I:C groups at the site of immunization, as well as innate immune cell recruitment and activation. This study suggests MPL+Poly I:C as a potential CMI-inducing vaccine adjuvant candidate.

**Abstract:**

Induction of antigen-specific cell-mediated immunity (CMI), as well as humoral immunity, is critical for successful vaccination against various type of pathogens. Toll-like receptor (TLR) agonists have been developed as adjuvants to promote vaccine efficacy and induce appropriate immune responses. Monophosphoryl lipid A (MPL); a TLR4 agonist, and Poly I:C; a TLR3 agonist, are known as a strong immuno-stimulator which induce Th1 response. Many studies proved and compared the efficacy of each adjuvant, but no study has investigated the combination of them. Using ovalbumin protein antigen, MPL+Poly I:C combination induced more effective antigen-specific CMI response than single adjuvants. Production of inflammatory cytokines, recruitment of innate immune cells and antigen-specific CD4/CD8 memory T cell at the immunized site had been significantly enhanced by MPL+Poly I:C combination. Moreover, MPL+Poly I:C combination enhanced ovalbumin-specific serum IgG, IgG1, and IgG2c production and proliferative function of CD4 and CD8 T cells after in vitro ovalbumin peptide stimulation. Taken together, these data suggest that the combination of MPL and Poly I:C has a potency as a CMI-inducing vaccine adjuvant with synergistically increased effects.

## 1. Introduction

Many studies have shown the importance of antigen specific cell-mediated immunity (CMI) and humoral immunity for successful vaccination against various types of pathogens [1,2]. Many vaccine adjuvants have been developed and included in vaccine regimens to enhance the efficacy of the vaccine and induce the appropriate immune responses [3]. An adjuvant is an immune-stimulator, which induces innate immunity by activating the pathogen recognition receptors (PRRs), such as the Toll-like receptors (TLRs). Activation of PRRs leads to the production of pro-inflammatory cytokines, the recruitment and maturation of antigen-presenting cells (APCs), modulation of the adaptive immunity by influencing the T and B cell responses, and induction of memory cells [4,5]. These memory cells consist of two main subsets: CD62L^high^CCR7^high^ central memory T cells (Tcm), and CD62L^low^CCR7^low^ effector memory T cells (Tem). Tcm cells are prevalent in lymph nodes and become highly proliferative after an antigen re-encounter, while Tem cells are predominantly found in the circulation and can be easily recruited to the sites of inflammation [6]. The memory T cells remaining after infections provide strong and long-term protection against re-infection [7,8].

Aluminum salts (alum) were the first adjuvants licensed for human vaccines and have been used for decades. They can be used to achieve high antibody responses, along with T helper (Th)-2 immune responses; however, they have limited capacity for the stimulation of CMI and induction of Th1 responses, showing poor protection against intracellular pathogens [9]. Thus, induction of CMI, especially with Th1 responses, has become a major hurdle in the development of vaccines [10]. Few adjuvants, including alum, MF59^®^ by Novartis, AS03 (squalene-based oil-in-water emulsions), and AS04 (alum + MPL) by Glaxo–Smith–Kline have been licensed for use in human vaccines [11]. Adjuvants systems (AS) refer to various combinations of adjuvants, to stimulate immune responses via the activation of several PRRs [12]. AS01 (a combination of MPL + QS-21 + Liposome) [13], AS02 (a combination of MPL + QS-21) [14] are under clinical trial and studies examining the effect of various adjuvants combinations are on-going [15,16]. Recently, a combination of MPL and CpG, a TLR9 agonist, immunized with influenza split vaccine elicited strong antigen-specific Th1 immune responses and induced protective efficacy against homosubtypic and heterosubtypic influenza infections [17,18].

MPL, a TLR4 agonist, is a detoxified lipopolysaccharide (LPS), which has been proved to be an effective adjuvant in several studies [19]. Poly I:C is a synthetic double-stranded (ds) RNA, a mimic of viral dsRNA. It activates several PRRs including TLR3, retinoic acid-inducible gene 1 (RIG-I) and melanoma differentiation-associated gene 5 (MDA5). Activation of these receptors is important in inducing anti-viral immune responses [20]. By activating various signaling pathways simultaneously, Poly I:C elicits strong Th1 and CD8^+^ T cell immunity [21,22]. Given these properties, we hypothesized that the combination of MPL and Poly I:C might have synergic effects in improving the CMI response with a strong memory-T-cell response.

In this study, we investigated the effects of combination of MPL and Poly I:C on immune responses to ovalbumin (OVA) protein antigen, at a relatively low dose (1 μg and 10 μg, respectively) [23,24]. 

## 2. Materials and Methods

### 2.1. Animals and Reagents

Six-to-eight-week-old female C57BL/6 and Balb/c mice were purchased from Orient Bio and maintained at the Jeju National University Animal Facility. All mouse experiments were performed according to the guidelines of the Jeju National University approved Institutional Animal Care and Use Committees (IACUC) protocol (protocol number 2020-0050). MPL and Poly I:C were purchased from InvivoGen. OVA protein was purchased from Sigma-Aldrich and OVA peptides, OVA_257–274_ and OVA_323–339_, were purchased from GenScript. All reagents were prepared according to the manufacturer’s instructions.

### 2.2. Immunization

**Figure 1:**
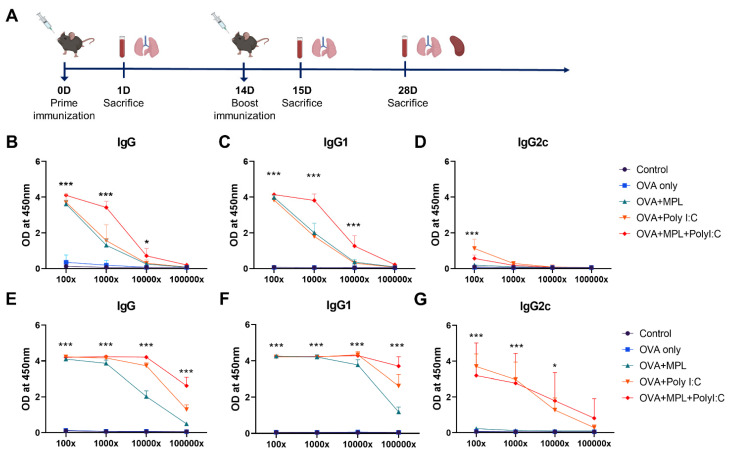
Immunization schedule and sera OVA-specific antibody levels after immunizations. (**A**) A scheme of immunization and sample collection schedule. (**B**–**G**) OVA-specific antibody levels in the immune sera were measured by ELISA. The sera were taken at 2-weeks after each prime (**B**–**D**) and boost (**E**–**G**) immunization. All result were shown in mean ± SEM. For statistical analysis, two-way ANOVA and Tukey’s post-multiple comparison tests were performed. * *p* < 0.05; and *** *p* < 0.001 compared with OVA group.

To investigate the efficacy of the adjuvants, MPL, Poly I:C, and MPL+Poly I:C, with OVA protein to induce the cellular and humoral immune responses and antigen-specific memory responses, immunizations were performed using the strategy shown in Figure 1A (15 mice per group). Briefly, the C57BL/6 mice were immunized intranasally with adjuvant combinations twice (prime and boost) at 2-week intervals. In previous studies, 10 to 500 μg of MPL and 50 to 1000 μg of Poly I:C have been used [23,24,25,26]. To decrease safety issues, immunization doses of each adjuvant more than five times lower were used in this study (1 μg/mouse of MPL; 10 μg/mouse of Poly I:C). The composition of each group was as follows: OVA-only (10 μg/mouse of OVA protein), MPL-adjuvanted (10 μg/mouse of OVA protein and 1 μg/mouse of MPL), Poly I:C-adjuvanted (10 μg/mouse of OVA protein; 10 μg/mouse of Poly I:C), and MPL+Poly I:C-adjuvanted (10 μg/mouse of OVA protein; 1 μg/mouse of MPL; 10 μg/mouse of Poly I:C); and the total volume of immunization was 50 μL/mouse. The control group received 50 μL of phosphate-buffered saline (PBS) intranasally. During the immunizations, the mice did not show any side effects, i.e., body weight loss. The mice were sacrificed at three time-points to collect samples: one day after prime immunization (Prime 1D), one day after boost immunization (Boost 1D), and 2 weeks after boost immunization (Boost 2w). The immune sera were collected at 2 weeks after each immunization. The bronchoalveolar lavage (BAL) fluid (BALF) and lung samples were collected at every time-point. Spleen and bone marrow samples were collected at Boost 2w.

### 2.3. Sample Preparation

Sera were taken by centrifugation of blood collected from the caudal vena cava. BALF was collected by inserting 18-gauge catheter into the trachea and washing the airway twice with 650 μL of PBS. After centrifugation, the supernatants were stored at −80 °C until cytokine enzyme-linked immunosorbent assay (ELISA) and the cell pellets were resuspended with 1 mL of PBS containing 2% fetal bovine serum (FBS) (FACS buffer) for flow cytometry. To acquire the lung and spleen cells, the tissues were mechanically disrupted, filtered by using a 100 μm cell strainer, and centrifuged. The supernatants were stored at −80 °C for cytokine ELISA. After red blood cells (RBCs) lysis, the cell pellets were resuspended with PBS and filtered by a 40 μm cell strainer for further analysis. Bone marrow cells were collected from the femur and tibia of the mice, as previously described [27].

### 2.4. Serum Antigen (Ag)-Specific Antibody ELISA

To measure Ag-specific antibody levels in the serum, serially diluted sera were then added to OVA-coated ELISA plates (400 ng/well) after blocking. Then, horseradish peroxidase (HRP)-labeled anti-mouse immunoglobulin (Ig) G, G1, and G2c secondary antibodies were used to detect the Ag-specific IgG in the serum. Tetramethylbenzidine substrate solution was used as the substrate, and the reaction was stopped by sulfuric acid. The optical density was measured at 450 nm wavelength. 

### 2.5. Cytokine ELISA

Cytokines in the BALF and lung extracts from the immunized mice at different time-points were measured using tumor necrosis factor (TNF)-α, interleukin (IL)-6 Mouse Uncoated ELISA Kit (Invitrogen), IL-12 p40, and interferon (IFN)- γ DuoSet ELISA kit (R&D system).

### 2.6. Memory B Cell Response

To measure the antigen-specific antibody production and memory B cell response, the cell culture plates were coated with the OVA protein (400 ng/well) overnight. The plates were washed and blocked with 10% complete media (200 μL/well) for 1 h at room temperature, before adding the cells. Spleen and bone marrow cells, harvested from the Boost 2w mice, were seeded at a density of 2 × 10^6^ cells/mL onto the plates and incubated at 37 °C for 7 d and 1 d, respectively. Anti-mouse IgG antibodies were used to detect Ag-specific antibodies produced by the cells.

### 2.7. Flow Cytometry

In Prime 1D and Boost 1D, the lung and BAL cells were harvested and stained with fluorophore-labeled antibodies specific for anti-mouse CD45 (clone 30-F11), CD11b (clone M1/70), CD11c (clone N418), F4/80 (clone BM8), Ly6c (clone AL-21), MCH class II (clone I-A/I-E), and Live/dead (L/D) to investigate inflammatory cell recruitment. In Boost 2w, to evaluate the memory T cell response, the lung, BAL, spleen cells were stained with CD45 (clone 30-F11), CD3 (clone 17A2), CD4 (clone RM4.5), CD8a (clone 53-6.7), CD44 (clone IM7), CD62L (Clone MEL-14), and L/D. To evaluate the antigen-specific memory T cell proliferation, the lung and spleen cells were stained with carboxyfluorescein succinimidyl ester (CFSE) and then seeded at a density of 5 × 10^5^ cells/mL and 2 × 10^6^ cells/mL, respectively, in 96-well plates, with OVA peptide stimulation. Two types of mixed OVA peptides (OVA_257–264_ and OVA_323–339_) were used. After a 5-day culture, the cells were collected and stained with CD3 (clone 17A2), CD4 (clone RM4.5), and CD8a (clone 53-6.7). 

For cell phenotype staining, the harvested cells were blocked at their Fc receptors using anti-CD16/32 (clone 2.4G2) antibody after washing with the FACS buffer. The antibody cocktail was added to the cells and incubated for 30 min at room temperature in the dark. The data were acquired using the BD FACS DIVA program and analyzed using the FlowJo software. 

The phenotypes of the acquired cells were gated and analyzed [6,28,29], and the gating strategies were provided in Figure S1: alveolar macrophages: CD45⁺ CD11b⁻ CD11c⁺ F4/80^+^; neutrophils: CD45⁺ CD11b⁺ Ly6c^lo^ F4/80⁻; monocyte-derived macrophages: CD45⁺ CD11b⁺ Ly6c^high^ F4/80^+^; total DCs: CD45⁺ F4/80⁻ CD11c⁺ MHCII ^high^; CD4 naïve T cell: CD45^+^ CD3^+^ CD4^+^ CD44^−^ CD62L^+^; CD4 central memory T cell (Tcm): CD45^+^ CD3^+^ CD4^+^ CD44^+^ CD62L^+^; CD4 effector memory T cell (Tem): CD45^+^ CD3^+^ CD4^+^ CD44^+^ CD62L^−^; CD8 naïve T cell: CD45^+^ CD3^+^ CD8^+^ CD44^−^ CD62L^+^; CD8 Tcm: CD45^+^ CD3^+^ CD8^+^ CD44^+^ CD62L^+^; CD8 Tem: CD45^+^ CD3^+^ CD8^+^ CD44^+^ CD62L^−^.

### 2.8. Bone Marrow-Derived Dendritic Cells (DCs) and Macrophage Culture and Mixed Lymphocyte Reaction (MLR)

The bone marrow cells were cultured in the Roswell Park Memorial Institute (RPMI) 1640 medium containing 10% FBS and 1x antibiotic-antimycotic (complete media) supplemented with 20 ng/mL mouse granulocyte–macrophage colony stimulating factor (mGM-CSF) to enrich the bone-marrow-derived DCs, as described previously [18]. The floating cells were removed and the old media was replaced with the fresh complete media with mGM-CSF, every 2 d. To generate bone marrow-derived macrophages, mouse macrophage colony stimulating factor (mM-CSF) was added in the culture media [30].

In vitro allogeneic MLR assay was conducted to determine the capacity of APCs to induce non-specific T cell proliferation. DCs and macrophages were generated from the bone marrow cells of Balb/c mice. Immature DCs were collected and seeded at a density of 5 × 10⁵ cells/mL in 6-well plates, while the immature macrophages were seeded at a density of 1 × 10^6^ cells/mL. The cells were cultured for 2 d with MPL (0.05 μg/mL), Poly I:C (2 μg/mL), or MPL+Poly I:C (MPL, 0.05 μg/mL; Poly I:C, 2 μg/mL). Allogenic naïve lymphocytes were harvested from the spleen cells of C57BL/6 mice, as described previously [30] and stained with 2 μM CFSE at 37 °C for 10 min. After washing, the pellet was resuspended in 10% complete media with 1 mM sodium pyruvate, 1× non-essential amino acids, and 55 μM 2-mercaptoethanol. The CFSE-labeled lymphocytes and prepared DCs were then seeded at a density of 2 × 10⁵ cells/well and 1 × 10^4^ cells/well of U-bottom 96-well plates in 200 μL of culture media, so that the ratio of DCs to lymphocytes was 1:20. The macrophages were seeded at a density of 1 × 10^5^ cells/well, so that the ratio of macrophages to lymphocytes was 1:2. After 5 d co-culture, the cells were harvested, stained with CD3 (clone 17A2), CD4 (clone RM4.5), and CD8 (clone 53-6.7), and analyzed by flow cytometry to determine the T cell proliferation.

To evaluate the antigen-specific memory T cell proliferation, ex vivo MLR was performed. OVA pre-treated DCs and macrophages generated from bone marrow cells of C57BL/6 mice were used for stimulating memory T cells, by providing antigen. For ex vivo MLR, the spleen cells were harvested from the immunized C57BL/6 mice at 2-weeks post-boost immunization and co-cultured with OVA pre-treated DCs or macrophages for 5 d. Then, the levels of IFN-γ cytokine were measured from the culture supernatants by ELISA.

### 2.9. Statistical Analysis

All results are presented as the mean ± standard error of mean (SEM) and statistical significance was determined by one-way analysis of variance (ANOVA) followed by Tukey’s multiple comparison test. All data were analyzed using the Graphpad Prism software 9.2.0 (GraphPad Software Inc., San Diego, CA, USA). 

## 3. Results

### 3.1. Combination of MPL and Poly I:C Enhanced the OVA-Specific IgG Antibody Responses 

To evaluate the adjuvant effects of the combination of MPL and Poly I:C in vivo, C57BL/6 mice were immunized with OVA with or without MPL, Poly I:C, or MPL+Poly I:C intranasally twice (prime and boost) at 2-week intervals (Figure 1A). After 2 weeks of each immunization, sera were collected and OVA-specific antibodies were measured by ELISA (Figure 1B–G). The adjuvanted groups significantly enhanced OVA-specific antibody production, whereas OVA-only immunization did not induce any OVA-specific antibody responses, even after the boost immunization. The MPL+Poly I:C-adjuvanted group showed approximately 10-times-higher levels of OVA-specific IgG and IgG1 isotype antibodies in sera at 2-weeks post immunization compared with those in single-adjuvanted groups. After prime immunization, OVA-specific IgG2c was induced by Poly I:C adjuvant, but after boost immunization, both Poly I:C-adjuvanted and MPL+Poly I:C-adjuvanted groups showed similar OVA-specific IgG2c levels in sera.

### 3.2. Combination of MPL and Poly I:C Promoted the Induction of Initial Inflammatory Cytokines after Immunization

**Figure 2:**
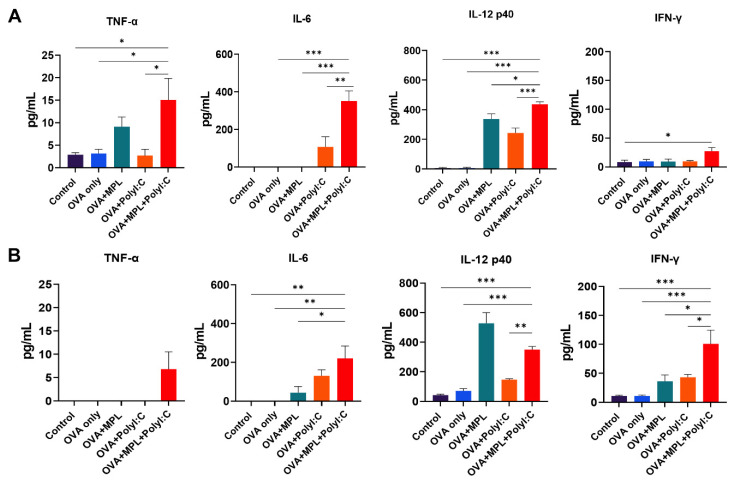
Cytokine production in lung tissue after prime and boost immunizations of mice. Lung extracts were harvested from the mice one day after prime immunization (**A**) and boost immunization (**B**). Levels of cytokine production from each sample were measured by ELISA. All results were shown in mean ± SEM. For statistical analysis, One-way ANOVA and Tukey’s post-multiple comparison tests were performed. * *p* < 0.05; ** *p* < 0.01; and *** *p* < 0.001 between the indicated groups.

Adjuvants have been used to induce inflammatory responses at the site of immunization to enhance adaptive immune responses. To evaluate the initial inflammatory responses after OVA immunization, with or without adjuvants, we measured cytokine levels in the lung extracts collected 1-day post-immunization. After the prime immunization (Figure 2A), OVA-only immunization did not induce any significant cytokine production in the lungs. MPL-adjuvanted immunization induced a moderate level of IL-12p40, but not TNF-α, IL-6, and IFN-γ. The Poly I:C-adjuvanted group induced IL-6 and IL-12p40 production. The combination of MPL and Poly I:C induced significantly higher levels of TNF-α, IL-6, IL-12p40, and IFN-γ production in the lungs compared with the OVA-only or single-adjuvanted groups. The patterns of cytokine production were maintained after the boost immunization (Figure 2B), so that the MPL+Poly I:C adjuvanted group showed significantly higher TNF-α, IL-6, IL-12p40, and IFN-γ production in the lung. These data suggest that the MPL+Poly I:C combination elicited a strong initial inflammatory immune responses at the site of immunization. 

### 3.3. OVA Immunization with the MPL and Poly I:C Combination Adjuvant Recruited Inflammatory Cells to the Site of Immunization

**Figure 3:**
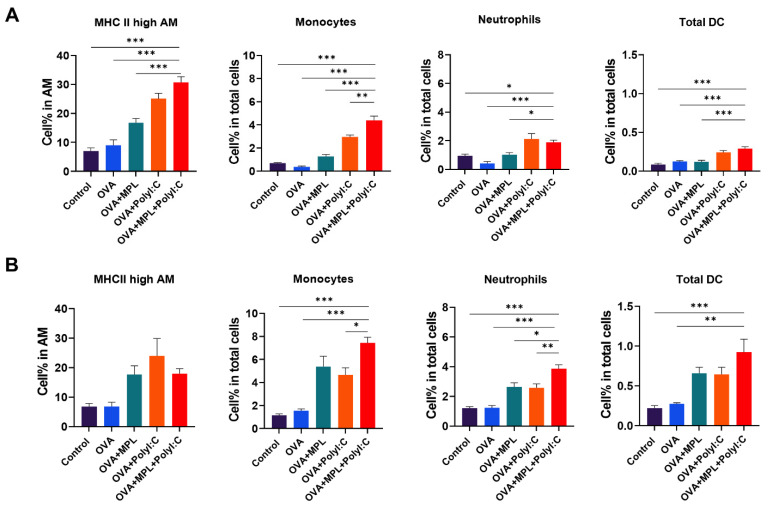
APC recruitment in Lungs after immunization. Lung cells were harvested from the mice one day after prime immunization (**A**) and boost immunization (**B**)**.** Cell phenotypes were analyzed by flow cytometry. All results were shown in mean ± SEM. For statistical analysis, One-way ANOVA and Tukey’s post-multiple comparison tests were performed. * *p* < 0.05; ** *p* < 0.01; and *** *p* < 0.001 between the indicated groups.

To evaluate the cell-recruiting effects of the MPL+Poly I:C combination at the site of immunization, we harvested lung cells from the immunized mice at day 1 post-prime and -boost immunizations. Cell phenotypes were determined using multicolor flow cytometry (Figure 3). OVA-only immunization did not induce cell recruitment in the lungs. After prime immunization (Figure 3A), Poly I:C adjuvanted immunization induced the recruitment of monocytes, neutrophils, and total DC populations in the lungs. The frequencies of monocytes and total DCs were significantly increased by the combination of MPL and Poly I:C. In addition, the activation of alveolar macrophages, which was measured by the expression levels of MHC class II molecules, was significantly enhanced by Poly I:C and MPL+Poly I:C-adjuvanted groups. These data suggested that Poly I:C effectively enhanced innate cell recruitment at the site of immunization, and the recruitment of antigen-presenting cells, such as monocytes and DCs, was synergistically increased by MPL supplementation with Poly I:C after the prime immunization (Figure 3A). After the boost immunization, the MPL-adjuvanted group also exhibited similar enhanced innate cell recruitment to those of the Poly I:C-adjuvanted group, and more innate cell recruitment was observed in the MPL+Poly I:C-adjuvanted group (Figure 3B). Compared to the prime immunization, 1.6-to-3 times more innate immune cells were recruited to the lungs after the boost immunization.

### 3.4. Adjuvants Promote In Vitro APC Activity to Proliferate Lymphocytes

**Figure 4:**
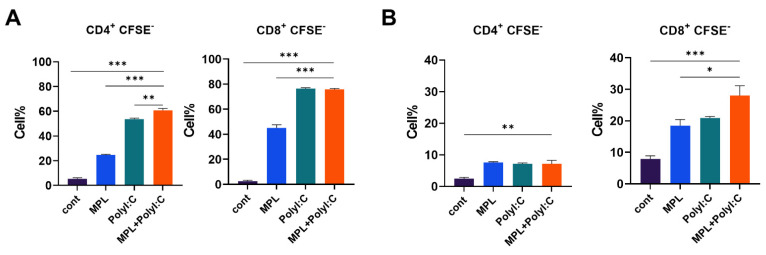
T cell proliferation after co-culture with adjuvant-pre-treated DCs and macrophages. DCs and macrophages generated from bone marrow cells were pre-treated with MPL, Poly I:C, or MPL+Poly I:C for 2 days. Allogeneic lymphocytes were harvested from spleen tissue. CFSE-labeled lymphocytes and pre-activated DCs (**A**) or macrophages (**B**) were co-cultured for 5 days. T cell proliferation was determined by flow cytometry. All results were shown in mean ± SEM. For statistical analysis, One-way ANOVA and Tukey’s post-multiple comparison tests were performed. * *p* < 0.05; ** *p* < 0.01; and *** *p* < 0.001 between the indicated groups.

To evaluate the APC functions stimulated by the adjuvants, MLR assay was performed. Bone marrow-derived DC and macrophages were stimulated with MPL, Poly I:C, or MPL+Poly I:C for 2-days, and then co-cultured with allogeneic naïve lymphocytes harvested from spleen. Poly I:C and MPL+Poly I:C treated DCs elicited significant CD4 and CD8 T cell proliferation (Figure 4A). MPL, Poly I:C, and MPL+Poly I:C-stimulated macrophages showed enhanced CD4 T cell proliferation than the control macrophages, and macrophages stimulated by MPL+Poly I:C promoted CD8 T cells significantly compared with other groups (Figure 4B). 

### 3.5. Effects of the Adjuvants on the Memory T Cell Population and Proliferation of Antigen-Specific T Cells 

**Figure 5:**
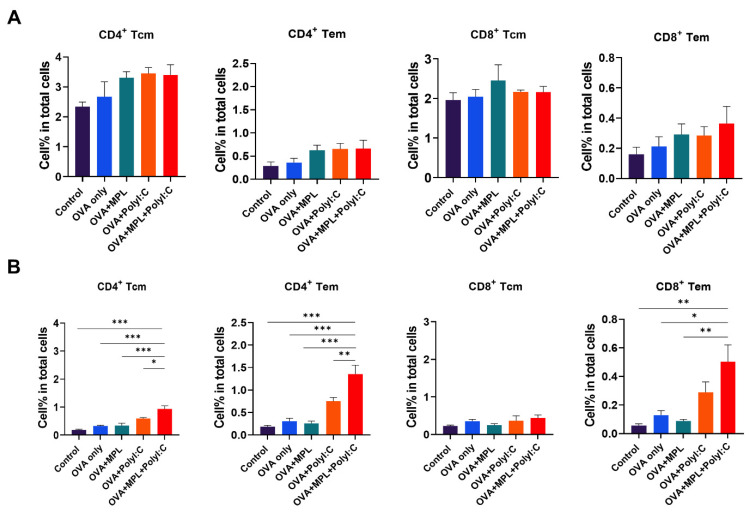
Memory T cell frequencies after immunization. The Tcm and Tem frequencies of CD4 and CD8 T cells in lungs (**A**) and spleens (**B**) after immunization were determined by flow cytometry. Lung and spleen cells were harvested from the mice 2 weeks after boost immunization. All results were shown in mean ± SEM. For statistical analysis, One-way ANOVA and Tukey’s post-multiple comparison tests were performed. * *p* < 0.05; ** *p* < 0.01; and *** *p* < 0.001 between the indicated groups.

To investigate memory T cell responses in the lung and spleen, cells were harvested from the mice at 2-week post-boost immunization. There was no significant difference between groups in memory T cell populations in spleen, but still increasing trends of memory T cell populations were observed in adjuvanted groups compared with those of the control or OVA-only groups (Figure 5A). The percentages of Tcm and Tem populations in both CD4 and CD8 T cells in the lung were significantly increased by the combination of MPL and Poly I:C, while single-adjuvanted groups did not induce a significant increase compared with the OVA-only group (Figure 5B). 

**Figure 6:**
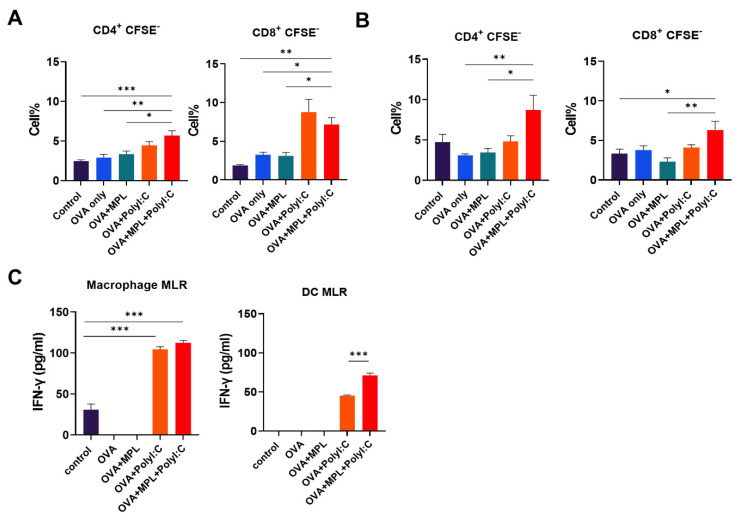
Antigen-specific memory T cell proliferation and cytokine production. Lung (**A**) and spleen (**B**) cells were harvested from the immunized mice 2 weeks after boost immunization. CFSE-labeled cells were cultured with OVA peptide for 5 days and T cell proliferation was determined by flow cytometry. (**C**) The spleen cells from the immunized mice were harvested at 2 weeks post-boost immunization and co-cultured with OVA pre-loaded macrophages or DCs for 5 days. IFN-γ secretion was measured by ELISA. All results were shown in mean ± SEM. For statis-tical analysis, One-way ANOVA and Tukey’s post-multiple comparison tests were performed. * *p* < 0.05; ** *p* < 0.01; and *** *p* < 0.001 between the indicated groups.

To investigate the OVA-specific memory T cell responses, T cell proliferation and cytokine production were measured after OVA restimulation. The antigen-specific T cell proliferation of lung and spleen cells was stimulated by OVA peptide treatment (Figure 6A,B). The antigen-specific CD4 T cell proliferation in the lung was induced in the MPL+Poly I:C-adjuvanted group. The antigen-specific CD8 T cell proliferation in the lung was similarly increased by Poly I:C and the combination of MPL and Poly I:C (Figure 6A). The MPL+Poly I:C-adjuvanted group showed enhanced Ag-specific CD4 and CD8 T cell proliferation after in vitro OVA peptide stimulation (Figure 6B). To further investigate the function of Ag-specific T cells after immunization, spleen cells were harvested from immunized mice and co-cultured with OVA-loaded macrophages or DCs. IFN-γ cytokine production from co-cultured cells was then evaluated. OVA-specific IFN-γ production was improved by Poly I:C and the combination of MPL and Poly I:C immunized spleen cells after co-culture with OVA-loaded macrophages and DCs (Figure 6C). These data implied that Poly I:C could efficiently induce memory T cell responses, and the responses can be greatly enhanced by combination with MPL, showing numerical and functional improvement of the memory T cells, especially in the immunized site.

### 3.6. Combination of MPL and Poly I:C Enhanced the Antigen-Specific Memory B Cell Responses

**Figure 7:**
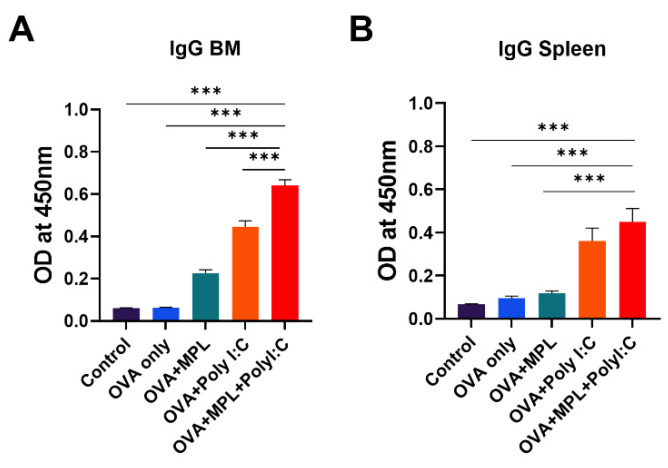
OVA-specific IgG production of bone-marrow cells and spleen cells from the immunized mice. BM cells (**A**) and spleen cells (**B**) were harvested from the immunized mice 2 weeks after boost immunization and cultured with OVA protein for 1 days or 7 days, respectively. Then OVA-specific IgG production was measured by ELISA. All results were shown in mean ± SEM. For statistical analysis, One-way ANOVA and Tukey’s post-multiple comparison tests were performed. *** *p* < 0.001 between the indicated groups.

After immunization, B cells are stimulated and differentiated into antibody-producing plasma cells and memory B cells. The plasma cells migrate to the bone marrow and keep producing antibodies, whereas the memory B cells stay in lymphoid tissues and are activated by antigen stimulation [31]. We harvested bone marrow cells and spleen cells from the immunized mice after 2 weeks of boost immunization to evaluate the ex vivo capacity of plasma cells and memory B cells to produce OVA-specific antibodies (Figure 7). Poly I:C and MPL+Poly I:C adjuvants significantly promoted both antibody-producing cells in the bone marrow and memory B cells in the spleen; in particular, the combination adjuvant showed synergistic effects on inducing antibody-producing cell activity. These data suggest that the MPL+Poly I:C combination elicited strong antibody-producing cells and memory B cell responses, as well as memory T cells. 

## 4. Discussion

Vaccination is the best way to protect life against invading pathogens. Most vaccine studies have focused on B cell responses, including neutralizing antibodies, but protection against pathogens such as viruses requires cell-mediated immune responses. Therefore, developing an effective vaccine adjuvant, antigen-specific T cell responses needs to be evaluated in addition to B cell responses and safety [32]. 

In addition to antigen-specific memory T cell responses, helper T cells affect antibody production via T-B cognition and cytokine production. Th1 immune responses mainly produce IFN-γ and induce IgG2c antibody production, while Th2 secretes IL-4 and elicits IgG1 antibody production [33]. In this study, we examined the effects of MPL and Poly I:C combination on the induction of antigen-specific T cell responses as vaccine adjuvant candidates. MPL stimulates the TLR4 signaling pathway, which elicits initial inflammatory responses via the TRIF–TRAM pathway [34,35]. It is known to be a safe immuno-stimulator to induce Th1 immune responses [36], but MPL-adjuvanted OVA immunization induced poor OVA-specific IgG2c antibody production in this study, suggesting Th2-skewed responses by MPL. However, the TLR3, RIG-1, and MDA5 signaling pathway stimulated by Poly I:C, activates the nuclear factor kappa-light-chain-enhancer of activated B cells (NF-κB) and Iinterferon regulatory factor 3 (IRF-3); virus-mediated signaling, which directly enters the nuclear membrane and elicits fast antiviral responses [37]. In this study, Poly I:C induced more effective CMI responses, with higher IgG2c antibody responses compared with those of the MPL-adjuvanted group, showing results consistent with previous studies comparing adjuvant effects [24,38]. The combination of MPL and Poly I:C adjuvants enhanced the initial inflammatory responses at the immunized site, showing higher levels of inflammatory cytokines and recruiting more APCs to the lungs. Consequently, it induced stronger OVA-specific T cell and antibody responses at 2-weeks after boost immunization. Improved IgG, IgG1, and IgG2c production in sera and OVA-specific IgG production in spleen cells and bone marrow cells demonstrated that B cells and T cells were functionally improved by the MPL+Poly I:C combination. We found that the Poly I:C and MPL+Poly I:C adjuvants increased the CD4 T cell population and decreased the CD8 T cell population (data not shown). However, the population of memory T cell subsets had increased in both CD4 and CD8 T cells, maintaining the ratio of Tcm to Tem. In addition to the numerical increases, the proliferation capacity of memory T cells after in vitro OVA treatment was also improved by MPL+Poly I:C adjuvanted immunization. 

The Ag-presenting process of APCs, such as DCs or macrophages, is crucial to induce Ag-specific T cell immunity [39]. MPL and Poly I:C stimulated DC activation and upregulated the co-stimulatory molecule expression on DCs, which was critical to initiate T cell response [23,40]. Additionally, Poly I:C contributed to the antiviral response of macrophages by promoting the differentiation of type 1 macrophages and pro-inflammatory phenotype [41]. After 1-day post-immunizations, MPL was effective to induce IL-12p40 production from lung APCs, but other inflammatory cytokines were significantly increased in the MPL+Poly I:C adjuvanted groups (Figure 2). In addition, the frequencies of the activated alveolar macrophages and DCs were highly elevated by MPL+Poly I:C adjuvant (Figure 3). Our in vitro MLR data supported the potency of MPL and Poly I:C adjuvants in the activation of DCs and macrophages. MPL+Poly I:C-treated DCs induced high proliferation of CD4 T cells, and MPL+Poly I:C-treated macrophages induced high proliferation of CD8 T cells (Figure 4). The MPL+Poly I:C combination induced Ag-specific T cell immunity through various stimulation pathways on APCs and T cells, leading to successful induction of memory T cell responses as well as innate immune cell activation. 

Safety profiles are required to be evaluated during a new vaccine adjuvant’s development. The autoimmune/inflammatory syndrome (ASIA) is induced by adjuvants included in vaccine regimens and causes mild-to-severe clinical autoimmune conditions in patients [42]. Both MPL and Poly I:C used in this study are belong to TLR agonists so that they can activate and trigger the TLR signaling pathway. This may cause some levels of inflammation, either locally or systemically. However, more than 5-times-lower doses of MPL (1 μg/mouse) and Poly I:C (10 μg/mouse) were used in this study compared with other studies [23,24,25,26], which reduced the concern of the autoinflammation and excessive immune responses. Also, no side effects, such as body weight loss, discomfort, and cough, were not observed from the mice during overall experimental periods.

In summary, we demonstrated the distinct effects of an MPL and Poly I:C combination on the induction of antigen-specific T cell responses, as well as on better antibody production, and stronger APC stimulation. It might be an effective vaccine adjuvant for vaccines against intracellular pathogens, like viruses. The development of novel vaccines is in high demand, to provide protection against various viruses, such as influenza virus, respiratory syncytial virus, human immunodeficiency virus, and severe acute respiratory syndrome-coronavirus 2. Future studies are required to investigate the adjuvance efficacy of the combination of sn adjuvant with viral vaccine candidates, and also it is necessary to determine the protective efficacy of the adjuvanted vaccine candidates against the infection. 

## 5. Conclusions

To improve the efficacy of a vaccine and induce appropriate immune responses, novel vaccine adjuvants have been developed and investigated by many research groups and companies. However, despite much research for adjuvant development, very few vaccine adjuvants have been licensed to date. The MPL+Poly I:C combination adjuvant, at low doses with OVA antigen, successfully elicited antigen-specific antibody production, memory T cell responses as well as innate immune cell activations, without side effects, in a mouse model. This study demonstrated the potential of the MPL+Poly I:C as a CMI-inducing vaccine adjuvant, and further studies should apply the combination strategy with other vaccine antigens.

## Data Availability

The data presented in this study are available on request from the corresponding author.

## Supplementary Materials

The following are available online at https://www.mdpi.com/article/10.3390/biology10090908/s1, Figure S1: Gating strategy of flow cytometry.
