# Supplementary material for: Monophosphoryl Lipid A and Poly I:C Combination Adjuvant Promoted Ovalbumin-Specific Cell Mediated Immunity in Mice Model
Source: Biology (Basel). 2021 Sep 13;10(9):908. doi: 10.3390/biology10090908 (PMC8471534; doi:10.3390/biology10090908)
Supplement: Supplementary file 1 [file biology-10-00908-s001.zip › biology-1337694-supplementary.pdf]

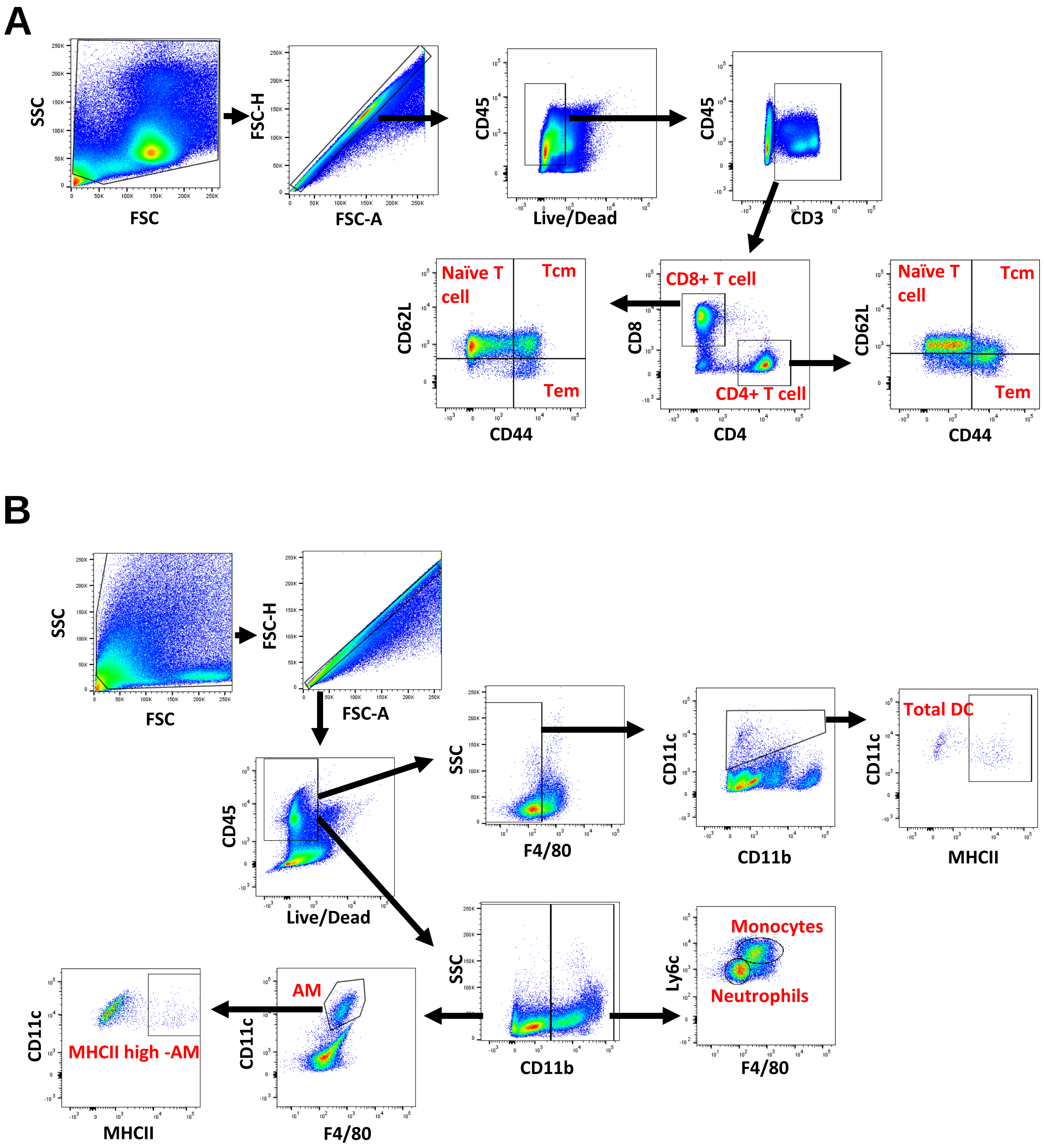

Supplementary Figure S1. Gating strategy of flow cytometry. (A) Memory T cell gating strategy. **(B)** Inflammatory cells and APCs gating strategy.
